# Editorial: Digitalization for precision healthcare

**DOI:** 10.3389/fpubh.2022.1078610

**Published:** 2022-12-02

**Authors:** Fidelia Cascini, Flavia Beccia, Francesco Andrea Causio, Natasha Azzopardi Muscat, Walter Ricciardi

**Affiliations:** ^1^Section of Hygiene and Public Health, Department of Life Sciences and Public Health, Università Cattolica del Sacro Cuore, Rome, Italy; ^2^Department of Health Services Management, Faculty of Health Sciences, University of Malta, Msida, Malta

**Keywords:** precision healthcare, Artificial Intelligence, COVID-19, public health, digitalization

Precision healthcare has the potential to transform medical practice, improve the quality of care and increase the sustainability of health systems. When based on the integration of data from multiple sources, including demographic, biological, behavioral, and environmental data, it can offer the best therapeutic approach to everybody as personalized medicine by delivering that also on time. A comprehensive data-driven approach can prevent the onset, progression, and recurrence of diseases at a large scale and shape the clinical pathways.

The creation of digital infrastructure and technologies to collect, analyse and connect electronic health and life-science data supports—now more than ever—the growth of precision healthcare. It has been, for instance, highlighted by Cascini, Santaroni et al. how data from Electronic Health Records (EHRs) can be used to design clinical pathways to improve the safety and quality of care, particularly in settings affected by healthcare workers shortages or equipment availability limitations. However, the current adoption of digital health tools and infrastructures is geographically variable and often missing an assessment, as expressed by Gentili et al. in a systematic review of the cost-effectiveness of digital interventions. This lacking approach to the digitalisation of the health sector has the effect of wasting resources with no improvement in care.

Undoubtedly, the pandemic stressed the health systems worldwide and acted as a boost in promoting the digitalisation of healthcare services. Dagliati et al. experimented with a predictive model based on EHRs to distinguish COVID-19 patient subgroups characterized by similar disease manifestations and clinical evolution. This model was applied to support organizational and clinical decision-making activities, setting up personalized treatments to improve patient outcomes and reduce healthcare costs.

Innovative solutions from the field of digital health can be used to investigate patient subgroups' responses to different patient journeys and to generate novel hypotheses regarding effective and setting-specific organizational, diagnostic, and therapeutic strategies. Sanmarchi et al. suggest implementing blockchain and other Distributed Ledger Technologies (DLTs) to increase patient engagement and improve the quality of healthcare organizational processes. By making data transactions safer and easily traceable, these technologies show increased potential to increase data sharing, which is crucial to ensure a personalized approach to health and integrate data from various devices and available medical tests, including genomic tests, hence improving the possibilities for public health surveillance and monitoring (1). These technologies also show their usefulness in ensuring the reproducibility of research and increasing the return on investment for research funders, editors, and publishers.

However, to increase the positive impacts of digitalisation investments on people's health, the extension of digital interventions should significantly allow the creation of a digitalised healthcare ecosystem. According to this, an overview of digital COVID-19 certificate initiatives, by Cascini, Causio et al. shows that the European experience of digitalised vaccination passes was based on the harmonization of technical specifications and standards, thus strengthening the control of the pandemic and allowing safe people mobility around the European Union.

Other promising applications of digital health for precision healthcare concerns the use of Artificial Intelligence (AI) tools in the fields of preclinical studies and clinical trials. AI-based solutions are promising to limit the waste of time supporting healthcare workers' tasks while increasing their accuracy (2). A scoping review by Cascini, Beccia et al. reveals that it is possible to promote the sustainability of clinical trials and the quality of their processes through the use of AI techniques, such as deep learning and natural language processing, deployed with the greatest advantages in clinical trial design and patients recruitment and matching (Cascini, Beccia et al.).

Digitalisation is then well-detectible and powerful in many contexts of the health sector, offering, from a public health perspective, the opportunity to drastically extend the accessibility, equity and sustainability of healthcare services. However, it should be noted that integrating these technologies in public health is coming at a slower pace than in other fields (3). All the papers collected within the Research Topic “*Digitalization for precision healthcare*” have the common focus of promoting new and innovative models to approach differently the complexity of healthcare systems. New guidelines and regulations are further emerging on the matter of the digitalisation of healthcare to embrace and address, at the same time, such new opportunities. The European Union is one of the main players in the field with the recent Artificial Intelligence Act and the Regulation Proposal on the European Health Data Space (4, 5). Nevertheless, it is crucial that, alongside the updating of the relevant legislation, digital tools are properly evaluated and adapted to the specific context of application and the existing infrastructures. The role of policymakers and healthcare managers will be determinant in transforming opportunities into realities.

## Author contributions

All authors listed have made a substantial, direct, and intellectual contribution to the work and approved it for publication.

## Conflict of interest

The authors declare that the research was conducted in the absence of any commercial or financial relationships that could be construed as a potential conflict of interest.

## Publisher's note

All claims expressed in this article are solely those of the authors and do not necessarily represent those of their affiliated organizations, or those of the publisher, the editors and the reviewers. Any product that may be evaluated in this article, or claim that may be made by its manufacturer, is not guaranteed or endorsed by the publisher.
